# Evaluation of hepatic lipogenesis and antioxidant status of broiler chickens fed mountain celery

**DOI:** 10.1186/s12917-018-1561-6

**Published:** 2018-08-13

**Authors:** Behnam Ahmadipour, Hossein Hassanpour, Fariborz Khajali

**Affiliations:** 1 Department of Animal Science, Shahrekord University, Shahrekord, 88186-34141 Iran; 2 Department of Basic Science, Shahrekord University, Shahrekord, 88186-34141 Iran

**Keywords:** Antioxidant, Chicken, Celery, Hepatic lipogenesis

## Abstract

**Background:**

Fatness is an unwanted side effect of genetic selection in broiler chickens. In this study, we introduce mountain celery powder as a feed supplement to suppress lipogenesis and improve antioxidant status in broiler chickens. Male broiler chicks (Ross 308) were fed a control diet or a diet that includes mountain celery (MC) at 7.5 gkg^−1^over 42 days.

**Results:**

Body weight gain and feed conversion ratio significantly (*P* < 0.05) improved in chicks fed MC. A highly significant down-regulation of genes involved in hepatic lipogenesis including acetyl CoA carboxylase (ACC), fatty acid synthase (FAS), malic enzyme (ME), and lipoprotein lipase (LPL) was observed in the liver of chickens fed MC*.* These birds, however, had greater compensatory upregulation in antioxidative genes SOD1 and catalase in the liver compared to the birds that received the control diet. Birds received MC had significantly lower level of lipid peroxidation (1.59 μmol/L serum malondialdehyde) compared to birds from the control group (3.57 μmol/L; *P* = 0.0024). Birds fed MC had significantly (*P* < 0.05) lower circulatory concentrations of triacylglycerols, cholesterol, and LDL but higher concentrations of HDL. Relative liver weight and abdominal fat deposition were significantly reduced by feeding MC*.*

**Conclusions:**

It can be concluded that feeding birds MC significantly suppresses hepatic lipogenesis by down-regulating key hepatic lipogenic enzyme genes and boosts antioxidant capacity by up-regulating hepatic antioxidantive genes.

## Background

Over the past several decades, intensive genetic selection in broiler chickens for rapid growth has led to their extraordinarily high growth rate. From 1957 to 2005, the body weight of broilers experienced a four-fold increase with a compounded annual growth rate of 3.30%. Concurrently, the 42-day feed conversion ratio (FCR) of chickens decreased by 2.55% each year over the same period [1]. The main side effect of such enormous growth rate is excessive fat accumulation in the body [2]. Modern broilers tend to mature more quickly and begin depositing fat at an earlier age. The chicken meat industry now faces the challenge of controlling excessive fat. Body fat in broiler chickens are generally not physiologically essential for body functions but impose a heavy burden on the antioxidant system [3] as majority of the fat is unsaturated, which is susceptible to oxidation [4]. Lipid peroxidation is a major cause of poor food quality and generation of off-odours and off-flavours. The incorporation of antioxidants is the most effective way to control the rate and extent of lipid peroxidation [5]. Therefore, dietary antioxidants in chickens play a pivotal role in protecting unsaturated fats as well as the bird’s body during digestion and metabolism, thereby the producion of high-quality meat.

Mountain celery (MC; *Kelussia odoratissima*; Umbelliferae family) is a rich source of polyphenols and flavonoids, and has antihyperlipidemic and antioxidant properties [6, 7]. Mountain celery is a self-growing plant, growing up to 200 cm in height and is found exclusively in Iran. Flavonoids and polyphenols are strong natural antioxidants with lipid-lowering capacity [5, 8]. The essential oil of MC is also a rich source of phthalides (37.3% of total bioactive components) [9], which are reported to be hypolipidemic, hypoglycemic, hypotensive, and antioxidant. The antioxidant potency of MC is has previously been described as comparable to α-tocopherol and butylated hydroxytoluene (BHT) [10]. Feeding MC to broiler chickens at 5 and 7.5 gkg^− 1^ resulted in a significant improvement in growth performance and was associated with lower rate of mortality from right ventricular hypertrophy [11]. Muscle relaxing and sedative effects of MC have been reported in mice [12]. The present study aimed to investigate antihyperlipidemic and antioxidant effects of MC in broiler chickens.

## Methods

### Birds and experimental facility

The experiment was conducted at the Poultry Research Center of Shahrekord University, Shahrekord, Iran (altitude 2100 m) in accordance to the recommendations of the Guide for the Care and Use Committee of Shahrekord University. All procedures were conducted in accordance with guidelines set out by Directive 2010/63/EU in Europe, and were approved by the Ethical Committee of Shahrekord University Research Council.

A total of 250 day-old male broilers (Ross 308; average body weight: 44.5 g ± 1.25) were obtained from Behjoojeh Co., Shahrekord, Iran. A total of 200 chicks within a range of 43 to 47 g (i.e within ±2 SD) were randomly selected and assigned to 10 pens, each with 20 chicks. All pens had similar initial weights. Five of these pens were randomly assigned to each treatment. No statistical power calculation was conducted prior to the study. The sample size was based on our previous experience with similar design. All pens were equipped with a bell dinker and trough feeder. All chicks were allowed free access to feed and water and were provided with 23 L:1D lighting program throughout the trial. The house temperature was conformed to the strain recommendations.

### Treatments

A control diet based on corn and soybean meal was formulated according to the NRC recommendations [13] for the starting (1 to 3 wk. of age) and growing (3 to 6 wk. of age) stages (Table 1). The experimental diet was prepared by substituting wheat bran with 7.5 gkg^− 1^ MC, a concentration of MC that has previously been described as the optimal level for broiler chickens [11]. Diets were provided in mash form. Mountain celery (the leaves and shoots) were cut, air-dried, and ground for use in the experimental diets. Chemical composition of the samples as gkg^− 1^ DM include: protein 90, dietary fiber 75, calcium 18, phosphorous 6, sodium 0.4, chloride 2.8, potassium 20, polyphenols 102, and flavonoids 12.2. The investigators were blinded to group allocation during the farm trial and laboratory analyses.

**Table 1:** Composition of the basal diet to which mountain celery (MC) added for broiler chickens during starter (1–21 days) and grower (22-42 days) stages

Item (% unless noted)	Starter (Control)	Starter (MC diet)	Grower (Control)	Grower (MC diet)
Corn	46.85	46.85	55.75	55.75
Soybean meal (44% CP)	37.50	37.50	32.90	32.90
Fish meal (60% CP)	3.65	3.65	1.00	1.00
Soy oil	7.50	7.50	6.00	6.00
Dicalcium phosphate	1.30	1.30	1.20	1.20
Oyster shell	1.45	1.45	1.50	1.50
Salt	0.35	0.35	0.30	0.30
DL-Methionine	0.15	0.15	0.10	0.10
L-Lysine	–	–	–	–
Mineral supplement^a^	0.25	0.25	0.25	0.25
Vitamin supplement^b^	0.25	0.25	0.25	0.25
Wheat bran^c^	0.75	–	0.75	–
Mountain celery	–	0.75	–	0.75
Calculated composition				
AMEn (kcal/kg)	3200	3200	3200	3200
CP	23	23	20.00	20.00
Met	0.50	0.50	0.40	0.40
Met + Cys	0.85	0.85	0.75	0.75
Lys	1.30	1.30	1.08	1.08
Thr	1.05	1.05	0.93	0.93
Arg	1.50	1.50	1.31	1.31
Ca	1.03	1.03	0.90	0.90
Available P	0.46	0.46	0.36	0.36
Na	0.18	0.18	0.16	0.16
Cl	0.27	0.27	0.29	0.29
K	0.91	0.91	0.92	0.92
Na + K-Cl (mEq/kg)	233	233	236	236

^a^Provided the following per kilogram of diet: vitamin A (*trans* retinyl acetate), 3600 IU; vitamin D3 (cholecalciferol), 800 IU; vitamin E (dl-α-tocopheryl acetate), 7.2 mg; vitamin K3, 1.6 mg; thiamine, 0.72 mg; riboflavin, 3.3 mg; niacin, 0.4 mg; pyridoxin, 1.2 mg; cobalamine, 0.6 mg; folicacid, 0.5 mg; choline chloride, 200 mg

^b^Provided the following per kilogram of diet: Mn (from MnSO4・H2O), 40 mg; Zn (from ZnO), 40 mg; Fe (from FeSO4・7H2O), 20 mg; Cu (fromCuSO4・5H2O), 4 mg; I [from Ca(IO3)2・H2O], 0.64 mg; Se (from sodium selenite), 0.08 mg

^c^*mountain celery* powder was replaced for wheat bran to provide the level of 7.5 g/kg

### Measurements

Following the procedures established by the Association of Official Analytical Chemists (AOAC) [14], the experimental diets were analyzed for crude protein (Method 976.06), crude fiber (Method 978.10), calcium (Method 910.01), phosphorous (Method 966.01), sodium (Method 929.03), chloride (Method 915.01), and potassium (Method 956.01). The total phenolic content was determined in MC using the Folin–Ciocalteu method. In brief, 0.5 ml of each sample was mixed with 2.5 ml of Folin- Ciocalteu’s phenol reagent and kept for 5 min at 37 °C. Then, 2 ml of saturated Na_2_CO_3_ (7.5%) was added and the mixture was brought up to 10 ml using deionized-distilled water. The mixture was maintained at room temperature in the dark for 120 min before measuring its absorbance at 765 nm against a reagent blank. Gallic acid equivalent (GAE) was used as the reference standard and total phenolic content was expressed as mg of GAE per gram of each essential oil on the dry basis. Total flavonoid content was measured using the aluminum chloride colorimetric assay. An aliquot (1 ml) of sample extracts or quercetin standard solutions (20, 40, 60, 80 and 100 μg/ml) was added to a 10 ml volumetric flask containing 4 ml of distilled water. Then, 0.30 ml of 5% NaNO_2_ was added, followed by the addition of 0.3 ml of 10% AlCl_3_ after 5 min. Subsequently, 2 ml of 1 M NaOH was added and the volume was brought up to 10 ml with distilled water. The absorbance was measured against a blank at 510 nm. The total flavonoid content was expressed as mg quercetin equivalents.

Body weight gain and feed intake were recorded and feed conversion ration (FCR) was calculated for the 1- to 21-day, 21- to 42-day, and 1- to 42-day periods. At 42 days of age, 10 birds per treatment were selected for blood collection and processing. The selected birds had body weights within ~ 5% of the average pen body weight. Blood samples (3 mL) were collected from the brachial vein and centrifuged at 2500×g for 10 min to obtain serum for the determination of cholesterol, triglycerides (TAG), low-density lipoproteins (LDL), high-density lipoproteins (HDL) and malondialdehyde (MDA). Total cholesterol, TAG, LDL, and HDL were measured using commercial laboratory kits following the manufacturer’s manuals (Pars Azmoon Kits; Pars Azmoon, Tehran, Iran). Serum MDA concentration, a biomarker of lipid peroxidation, was assayed following the method of Nair and Turner [15]. An aliquot of blood was also obtained on glass slides to prepare a blood smear to determine differential leukocyte count. The blood was stained using the May-Grunwald and Giemsa protocol, and 100 leukocytes, including granular (heterophils) and nongranular (lymphocytes) were counted to calculate the heterophil to lymphocyte ratio (H:L). All chemical reagents were obtained from Sigma–Aldrich Co. (Sigma–Aldrich Co., St. Louis, MO, USA).

After collecting blood, the birds were euthanized by CO_2_ inhalation and we collected live body weight, liver weight, and abdominal fat deposition. The livers were harvested and immediately frozen in liquid nitrogen and stored at − 70 °C for RNA analyses.

### Quantitative real-time PCR analysis

Total RNA was extracted from the liver using RNX-Plus reagent (Sinaclon Bioscience, Tehran, Iran) and homogenized in digestion buffer. The liver was homogenized in digestion buffer, then mixed with chloroform and centrifuged. Total RNA in the upper aqueous phase was precipitated with isopropanol, rinsed with 75% ethanol, then resuspended in DEPC-treated water. To remove residual DNA, the RNA was treated with DNase (Sinaclon Bioscience, Tehran, Iran). The RNA was measured using a spectrophotometer. Only RNA with an absorbance ratio (A260/A280) > 1.9 was used for the synthesis of cDNA. Total RNA was reverse transcribed into cDNA using PrimeScript ™ RT Reagent Kit (Takara Bio Inc., Japan). The reverse transcription mix was heated to 85 °C for 5 s to inactivate reverse transcriptase and to denature the RNA and then stored at − 20 °C.

The levels of acetyl CoA carboxylase (ACC, EC 6.4.1.2), fatty acid synthase (FAS, EC 2.3.1.85), malic enzyme (ME, EC 1.1.1.40), lipoprotein lipase (LPL, EC 3.1.1.34), superoxide dismutase 1 (SOD1, EC 1.15.1.1), catalase (CAT, EC 1.11.1.6) and β-actin transcripts were determined by real-time reverse transcriptase (RT)-PCR using SYBR® Premix Ex Taq™ II (TliRnase H Plus; Takara Bio Inc., Japan). We used β-actin as an endogenous standard to normalize the input load of cDNA among samples. Specific primers of the transcripts were designed with Primer-Blast (www.ncbi.nlm.nih.gov/tools/primer-blast/index.cgi?LINK_LOC = BlastHome) are detailed in Table 2. PCRs were carried out in a real-time PCR cycler (Rotor Gene Q 6000, Qiagen, USA) in three replicates for each liver sample. One microliter cDNA was added to 10 μl of SYBR® Premix Ex Taq II Mix with 1 μM of each specific primer (total volume of 20 μl). The thermal profile was 95 °C for 30 s, 40 cycles of 94 °C for 40 s, 64 °C for 35 s and 72 °C for 30 s. Fluorescence was measured at the end of each phase. Gene expression data were normalized to β-actin. Data were analyzed using LinRegPCR software version 2012.0 (Amsterdam, Netherland) to obtain the threshold cycle number and reaction efficiency [16]. Relative transcript levels and fold changes in transcript abundance were calculated using efficiency-adjusted Paffl methodology [17].

**Table 2:** Details of the primers used for quantitative real time PCR analysis of chicken mRNAs

Target	Target Primers	PCR product (bp)	Accession No.
β-Actin	F:5’-AGCGAACGCCCCCAAAGTTCT-3’	139	NM_205518.1
	R:5’-AGCTGGGCTGTTGCCTTCACA-3’		
FAS	F:5’-GCTGGCTACAGTGGTGGACT-3’	129	NM_205155.2
	R:5’-CCACCTCGAACCACCAAAGC-3’		
LPL	F:5’-CTCCGATCCCGAAGCTGAGATG-3’	106	NM_205282.1
	R:5’-CTGTCCAGGAACCAGGTAGC-3’		
ACC	F:5’-CAACGAGTCGGGCTACTACC-3’	146	J03541.1
	R:5’-GGTCCTTGGTCACGTATGGG-3’		
MAE	F:5’-GGACCTCGAAGCCTTCATCC-3’	77	AF408407.1
	R:5’-GCCAGGTGTATGAGTCAGCC-3’		
CAT	F:5′-TGGCGGTAGGAGTCTGGTCT-3′	112	NM_001031215.1
	R:5′-GTCCCGTCCGTCAGCCATTT-3′		
SOD1	F:5-CACTGCATCATTGGCCGTACCA-3′	223	NM_205064.1
	R:5-GCTTGCACACGGAAGAGCAAGT-3′		

Abbreviations: *FAS* fatty acid synthase, *LPL* lipoprotein lipase, *ACC* acetyl-coA carboxylase, *MAE* malic enzyme, *CAT* catalase, *SOD1* superoxide dismutase 1

### Statistical analysis

Results were analyzed by ANOVA using SAS (2007) software in a completely randomized design. Treatment means were separated by t-test. In the cases of sampling within pens, data were subjected to a nested design.

## Results

Mountain celery analysis shows remarkable contents of polyphenols (102 mg/g), flavonoids (12.2 mg/g) and phtalides, mainly ligustilides. Ligustilides accounted for 50% of total active products in the mountain celery.

Body weight gain and FCR responses were significantly (*P* < 0.05) improved in broilers when MC was included in diets during all feeding stages. However, feed intake only showed a significant decrease in the starter stage (Table 3).

**Table 3:** Effects of dietary levels of mountain celery on broiler growth performance

Variable	Control	Mountain celery (7.5 gkg^− 1^)	*P*-value
Feed intake (g/bird)			
1–21 days of age	1052 ± 8.4^a^	1007 ± 10.9^b^	0.0127
21–42 days of age	2873 ± 44.3	2839 ± 32.4	0.5499
1–42 days of age	3925 ± 36.1	3846 ± 28.5	0.1262
Weight gain (g/bird)			
1–21 days of age	664 ± 8.6^b^	704 ± 7.3 ^a^	0.0081
21–42 days of age	1334 ± 18.3^b^	1488 ± 7.1 ^a^	0.0001
1–42 days of age	1998 ± 10.3^b^	2172 ± 20.3 ^a^	0.0001
Feed conversion ratio			
1–21 days of age	1.58 ± 0.009^a^	1.44 ± 0.022^b^	0.0002
21–42 days of age	2.15 ± 0.008 ^a^	1.91 ± 0.028^b^	0.0001
1–42 days of age	1.96 ± 0.011 ^a^	1.77 ± 0.026^b^	0.0001

Means in the same row with different letters are significantly different

Values are mean ± SE (*n* = 5)

The expression of genes responsible for hepatic lipogenesis, including ACC, FAS, ME, and LPL, significantly down-regulated in birds fed MC (Table 4). In contrast, antioxidant genes including SOD1 and CAT have been highly over-expressed in broilers fed MC (Table 4).

**Table 4:** Effect of dietary mountain celery on lipogenic and antioxidant gene expression in broiler chickens measured at 42 days of age

Variable	Control	Mountain celery (7.5 gkg^− 1^)	*P*-value
Acetyl CoA carboxylase	0.025 ± 0.007^a^	0.003 ± 0.001^b^	0.0088
Fatty acid synthase	0.071 ± 0.018^a^	0.011 ± 0.002^b^	0.0049
Lipoprotein lipase	0.012 ± 0.004^a^	0.002 ± 0.001^b^	0.0189
Malic enzyme	0.029 ± 0.009^a^	0.002 ± 0.001^b^	0.0111
Superoxide dismutase 1	0.0006 ± 0.0001^b^	0.014 ± 0.002^a^	0.0001
Catalase	0.0005 ± 0.0001^b^	0.005 ± 0.001^a^	0.0007

^a,b,c^Means in the same row with different letters are significantly different

Values are relative quantitative PCR (Target/β-actin)

Values are mean ± SE (*n* = 10)

Broilers that received MC had significantly (*P* < 0.05) lower concentrations of total TAG, total cholesterol, LDL, malondialdehyde, and heterophil to lymphocyte ratio. Feeding MC caused a significant (*P* < 0.05) increase in HDL compared to the control group (Table 5).

**Table 5:** Effect of dietary mountain celery on serum, blood, and carcass variables in broiler chickens measured at 42 days of age

Variable	Control	Mountain celery (7.5 gkg^− 1^)	*P-*value
Total cholesterol (mg/dL)	126 ± 5.7^a^	109 ± 5.1^b^	0.0006
Total triacylglycerols (mg/dL)	92.5 ± 5.30^a^	78.6 ± 3.80^b^	0.0003
HDL (mg/dL)	30.6 ± 2.75	38.6 ± 5.0	0.0031
LDL (mg/dL)	34.4 ± 2.93^a^	28.3 ± 1.17^b^	0.0001
Malonedialdehyde (μmol/L)	3.57 ± 1.31^a^	1.59 ± 0.77^b^	0.0024
Heterophils to Lymphocyte (%)	0.84 ± 0.16^a^	0.59 ± 0.12^a^	0.0021
Abdominal fat deposition (%)	1.80 ± 0.27^a^	1.20 ± 0.15^b^	0.0001
Relative weight of liver (%)	2.25 ± 0.21^a^	1.87 ± 0.19^b^	0.0001

^a,b^Means in the same row with different letters are significantly different

Values are mean ± SE (*n* = 10)

## Discussion

In general, live performance of the broilers in this study was lower compared to the expected performance outlined in the strain catalog (ROSS 308). This is due to the fact that the birds reared at high altitude region (2100 m), where atmospheric oxygen concentration was only 15–16%. There is a direct relationship between broiler growth performance and atmoshpheric oxygen concentration [3]. Significant improvements in body weight gain and FCR associated with feeding birds MC are in line with results reported by Ahmadipour et al. [11]. Improved performance can be attributed to the naturally-occurring polyphenols in MC, which include flavonoids and non-flavonoids that exhibit a broad spectrum of beneficial biological properties such as growth-promoting, antioxidative, and immunocompetence [8, 18]. Dietary inclusion of MC was associated with down-regulation of ACC, FAS, ME, and LPL in the liver. In avian species, the liver is the principal site of lipogenesis with a capacity 20 times greater than adipose tissue of equal weight [19]. The pathways involved in fatty acid synthesis in birds involve ACC catalyzing the conversion of acetyl-CoA to malonyl-CoA, and FAS catalyzing the formation of palmitate from one molecule of acetyl-CoA and 7 molecules of malonyl-CoA [20]. ME catalyzes the oxidative decarboxylation of malate to pyruvate and carbon dioxide, simultaneously generating nearly all of 14 molecules of NADPH required for palmitate biosynthesis [21]. Upon the biosynthesis of palmitate, fatty acids are transported to adipose tissue as TAG in the VLDLs, where extracellular LPL catalyses the hydrolysis of TAG so that fatty acids can be incorporated into the adipose tissue [22]. Down-regulation of these four key lipogenic enzymes by MC strongly suggests antihyperlipidemic effects as reflected in reduced levels of cholesterol, TAG, and LDL. The results of our study strongly suggest a strong nutrigenomic effect of MC on lipogenesis in birds, which may be associated with flavonoids and phthalides. A report on transcription-factor pathways mediating nutrient–gene interactions indicated ER (estrogen receptor), NFkB (nuclear factor kB), and AP1 (activating protein 1) as transcription factors associated with flavonoids [23]. However, transcription factor associated with phthalide has yet to be determined. Mountain celery is a rich source of polyphenols (102 mg/g), flavonoids (12.2 mg/g) and phtalides, manily ligustilide.

Aqueous extract of celery has been reported to reduce total cholesterol and LDL in rats by lowering hepatic LPL activity [24] – an antihyperlipidemic effect linked with phthalides. The hypolipidemic effects of flavonoids have also been previously documented [25]. Bok et al. [26] indicated that polyphenols reduce the activity of HMG-CoA reductase, a key enzyme in the synthesis of cholesterol in mice. It has also been reported that polyphenols alter cholesterol homeostasis and increase LDL-receptor activity in human, which can increase LDL levels [27].

Stimulation of expression of SOD1 and CAT by MC suggests that the herb improves antioxidant defense system in birds, which could reduce levels of malondialdehyde (MDA). Previous research has shown that phtalides decrease MDA levels and increase the activities of the antioxidant enzymes such as glutathione peroxidise (GSH-Px) and SOD in mice [28]. Ligustilides (the major components of phtalides in MC) have been reported to increase the activities of GSH-Px and SOD, which can prevent oxidative stress [29]. MDA is a biomarker of lipid oxidation is an index of oxidative stress in fat tissues. Decreased oxidative stress in birds fed MC can be associated with a significant reduction in the H:L ratio, an index of stress in the chicken [30]. Taken together, feeding MC can suppress ROS production and alleviate oxidative stress in birds, and can be reflected by lower levels of MDA and H:L ratio.

Antihyperlipidemic effects of MC include reduced deposition of fat in the abdominal cavity. Abdominal fat is a crucial component of lipogenesis in birds because it grows more rapidly compared to other adipose tissues and is highly correlated to total body fat content in avian species. As it is easy to dissect and weigh, abdominal fat weight has been used extensively as an estimator of total body fat content in the chicken [31]. Reduction in abdominal fat deposition in birds fed MC indicates the strong antihyperlipidemic potency of the herb. Reduction in the proportion of liver weight to live body weight in chickens fed MC was in line with decreased lipogenesis associated with reduced abdominal fat. As the liver is the principal site of lipogenesis in chickens [19], a decline in its relative weight can account for lower lipogenesis associated with dietary inclusion of MC.

## Conclusion

Feeding MC (*Kelussia odoratissima*) to broiler chickens led to a significant decrease in the expression of lipogenic enzymes and a significant increase in the expression of antioxidant enzymes, suggesting that this medicinal herb has potential to produce high quality low-fat chickens. These nutrigenomic effects are attributed to the phthalides, flavonoids, and polyphenols naturally in MC.

## Abbreviations

ACC: Acetyl CoA carboxylase
cDNA: Complementary DNA
DEPC-reated water: Diethylpyrocarbonate-treated water
FAS: Fatty acid synthase
H:L: Heterophil to lymphocyte
HDL: High-density lipoproteins
LDL: Low-density lipoproteins
LPL: Lipoprotein lipase
MC: Mountain celery
MDA: Malondialdehyde
ME: Malic enzyme
PCR: Polymerase chain reaction
TAG: Triacylglycerols
